# Progression of retinal pigmentation mimicking unilateral retinitis pigmentosa after bilateral pars planitis: a case report

**DOI:** 10.1186/s12886-018-0814-2

**Published:** 2018-09-10

**Authors:** José I. Vela, Ivanna Marcantonio, Jesús Díaz-Cascajosa, Jaume Crespí, José A. Buil

**Affiliations:** 1 Department of Ophthalmology, Hospital de la Santa Creu i de Sant Pau, Barcelona, Spain; 2 Department of Ophthalmology, Institut Condal d’Oftalmología, Barcelona, Spain

**Keywords:** Pars planitis, Pigmentation, Pseudoretinosis, Unilateral retinitis pigmentosa

## Abstract

**Background:**

To report our findings in a young patient with unilateral retinitis pigmentosa (RP)-like appearance who developed pigmentary changes in his left retina after an episode of bilateral pars planitis.

**Case presentation:**

A 17-year-old man presented with 6 months of blurry vision in both eyes. He was diagnosed with bilateral pars planitis. Progressive, intraretinal bone crepuscule pigmentation developed in his left retina during the following three months. An electroretinogram showed subnormal response only in the left eye, suggesting the diagnosis of unilateral pseudoRP.

**Conclusion:**

An inflammatory disease like pars planitis can accelerate the pigmentation of the retina and mimic a RP in young patients. Causes of pseudoRP may be considered, especially in those rare cases with unilateral affection.

## Background

Retinitis pigmentosa (RP) refers to a group of inherited diseases causing retinal degeneration. It is a major cause of visual disturbance and blindness in all age groups. It usually comes with a bilateral, symmetric impairment of visual functions along with night blindness and gradually concentric loss of peripheral vision [1]. However, unilateral cases have been described as a rare manifestation form of RP [2].

Retinal pigmentary changes as coarse clumps in a “bone spicule” configuration are typically observed in RP, but fundus appearance depends on the stage of retinal degeneration. In the earliest stages, especially in young patients without symptoms, the fundus usually appears normal and the diagnosis may be delayed. When no pigmentation is observed in the fundus despite documented abnormalities of retinal cell function, the term RP *sine pigmento* has been used [3].

Some disorders as melanoma of the choroid can lead to retinal changes with pigment irregularities, mimicking RP [4]. Few inflammatory eye diseases like congenital infections have been described, causing a fundus appearance similar to RP. In all of these cases (lupus, Fuchs’ heterochromic iridocyclitis, syphilis, toxoplasmosis, cytomegalovirus, rubella, measles or diffuse unilateral subacute neuroretinitis) RP must be ruled out [5, 6].

We present a case of a young patient who quickly developed pigmentary changes in his left retina mimicking a unilateral RP after an episode of bilateral pars planitis.

## Case presentation

A 17-year-old man with no known past medical history, presented with 6 months of blurry vision in both eyes. He had no other ocular, medical, or surgical history.

Baseline visual acuity (VA) was 20/20 in the right eye (RE) and 20/63 in the left eye (LE). His intraocular pressure was normal (17/16 mmHg). Slit lamp examination revealed normal anterior structures and no anterior segment inflammation. A subclinical keratoconus was described in the topography study. 1+ cells in the vitreous (graded on a scale of 0+ to 4+), snowballs and snow banking in the inferior pars plana as well as peripheral vasculitis were observed in both eyes (BE) on dilated fundus examination.

Serological tests for HIV, *Toxoplasma gondii*, *Borrelia burgdorferi* or *Treponema pallidum* were negative, as well as for antinuclear (ANA) and antineutrophil cytoplasmatic (ANCA) antibodies.

He was diagnosed with bilateral pars planitis. Optical coherence tomography (OCT) showed cystoid macular edema in the LE, so that sub-tenon injection of triamcinolone was performed in order to control inflammation, which was slowly reduced.

Subsequent follow-up visits showed improvement in vision to 20/25 in the LE, resolving progressively the inflammation of the posterior pole although 0.5+ cells were observed in the vitreous cavity. Then, no additional therapy was needed. Nonetheless, the patient complained of loss of visual field in his LE.

**Fig. 1:**
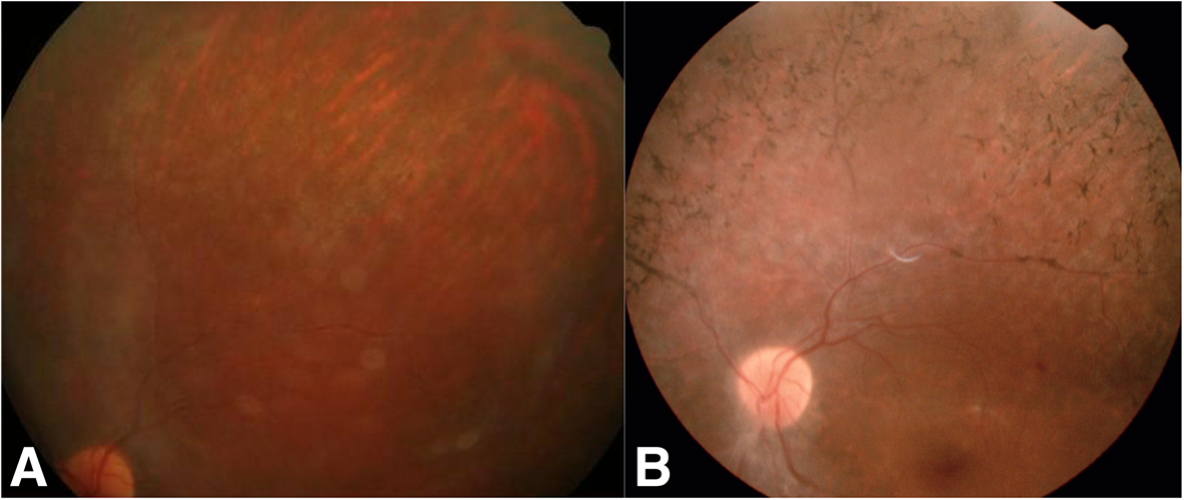
**a** Colour fundus photograph (superior field) of the left eye showing mild RPE mottling, blurred by vitreous cells. Retinal pigment deposits were not observed. **b** Pigmentation of the retina developed three months after onset

In-depth fundus examination showed a mild, diffuse, granular appearance of the retinal pigment epithelium (RPE) throughout the LE. Progressive, intraretinal bone crepuscule pigmentation developed during the following three months (Fig. 1). The RE showed no retinal pigmentation. Humphrey perimetry confirmed peripheric constriction of the visual field in the LE and no scotomas in the RE.

An electroretinogram (ERG) showed subnormal response only in the LE. The diagnosis of unilateral RP-like appearance was made.

## Discussion and conclusions

We report a rare case of pars planitis with progressive unilateral pigmentation of the retina. Unilateral RP (also called extremely asymmetrical RP) is a rare and controversial condition that has been described in the literature, but there are still doubts about its existence as an isolated clinical entity and its relation to RP. Some mechanisms purposed are the occurrence of genetic mosaics (mutations that affect only some of the cells) or somatic mutations (instead of germline mutations) [7, 8]. Since retinal dystrophies are usually bilateral due to their genetic background, a unilateral manifestation requires rule out other explanations.

The differential diagnosis for unilateral retinal pigment epithelium changes and peripheral field loss starts with asking for prior blunt trauma, retinal detachment or retained metallic intraocular foreign body. Treatment with antipsychotics from the group of phenothiazines can induce a peripheral pigmentary retinopathy [9]. Since our patient had no history of trauma or consumption of antipsychotics, this differential diagnosis could be excluded.

Some patients with cancer can develop antibodies against retinal antigens (antirecoverin antibodies), leading to a paraneoplastic syndrome called carcinoma-associated retinopathy or autoimmune retinopathy [10]. Since the autoantibodies circulate in the blood stream, BE are usually affected.

Retinal pigmentary changes can also occur in the context of past ocular infection or inflammatory eye disease. Syphilis, toxoplasmosis, cytomegalovirus, rubella, measles or diffuse unilateral subacute neuroretinitis (DUSN) must be ruled out [6]. They can occur uni or bilaterally, usually early in life. As described above, infections were excluded in our patient.

The patient was first diagnosed of pars planitis. Few publications of uveitis coexisting with RP have been reported, specially a form of non-granulomatous uveitis called Fuchs’ heterochromic iridocyclitis [11–14]. Dust-like particles in the vitreous are present in the great majority of young individuals with RP. These usually are fine, colorless particles comprising free melanin pigment granules, uveal melanocytes, EPR cells, and macrophage-like cells. These particles can be confused with vitreous cells or snowballs present in the intermediate uveitis. Particles in RP eyes are evenly distributed throughout the vitreous while the snowballs, larger in size, are observed at the bottom of the vitreous cavity. Observation of these small particles can be helpful in the diagnosis of early RP before pigmentary fundus changes are apparent. Our patient showed vitreous cells and snowballs in BE so that the observation of these dust-like particles in the LE was difficult.

The typical bone-spicule pigmentation represents migration of pigment into the interstitial spaces of the retina from disintegration of RPE cells. This pigment accumulation is greater in the surrounding spaces of the retinal vessels, producing perivascular pigmentary cuffing and spicule-shaped deposits. Almost all forms of RP go through a stage where no pigmentary changes exist in the retina. This stage may exist for decades before typical RP signs appear.

Pigmentation of the retina developed in only three months in our patient. Probably some grade of epithelitis developed in the left eye of our patient. We hypothesize that inflammation may accelerate the pass to the pigmentary stage. It is well known that inflammatory eye diseases like rubella, syphilis or DUSN present with pigmentary abnormalities within the retina [6]. Inflammation can promote a RPE cell reaction and stimulate the migration of the pigment into the retina. The pigment deposits are clumps or large patches of black pigment and can be associated to chorioretinal scars. Then, distribution of pigmentation due to inflammation is usually patched and typical bone-spicule pigment formations are uncommon.

In summary, an inflammatory process like pars planitis can accelerate the pigmentation of the retina. Asymmetrical RP must be ruled out in these unilateral RP-like appearance post-inflammatory changes.

## Abbreviations

BE: Both eyes
ERG: Electroretinogram
LE: Left eye
RE: Right eye
RP: Retinitis pigmentosa
RPE: Retinal pigment epitelium
VA: Visual acuity
